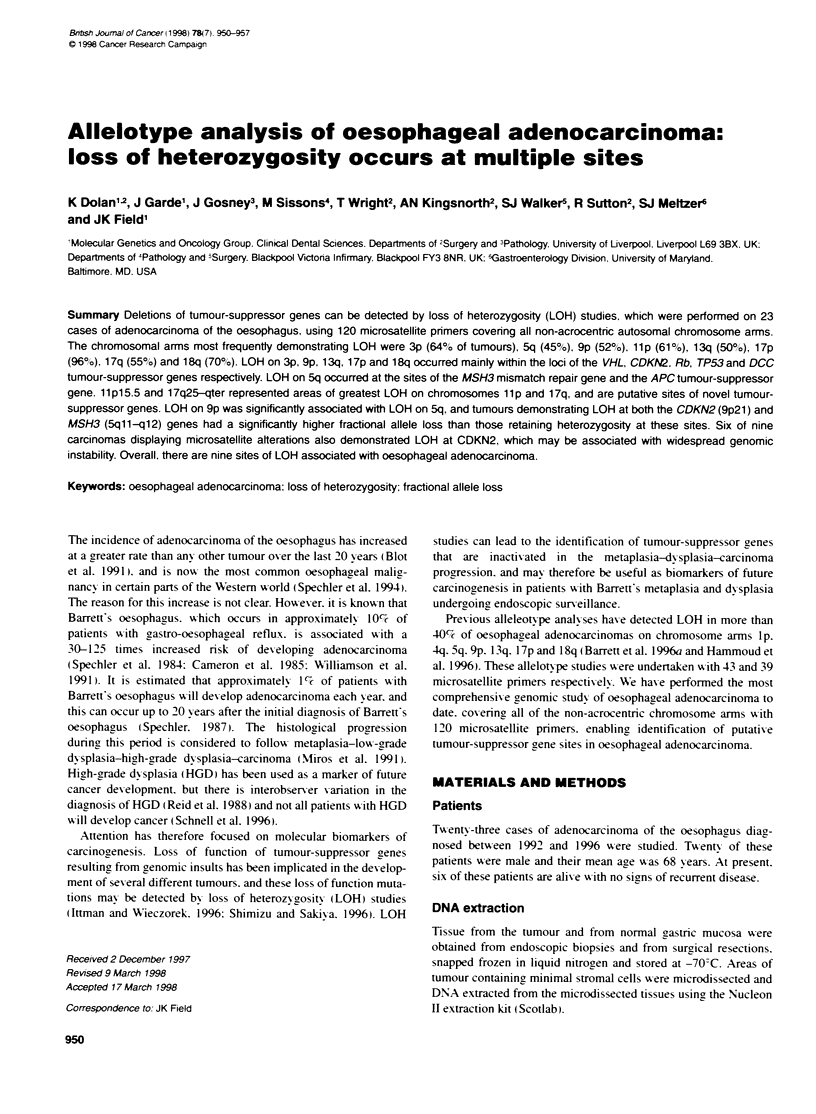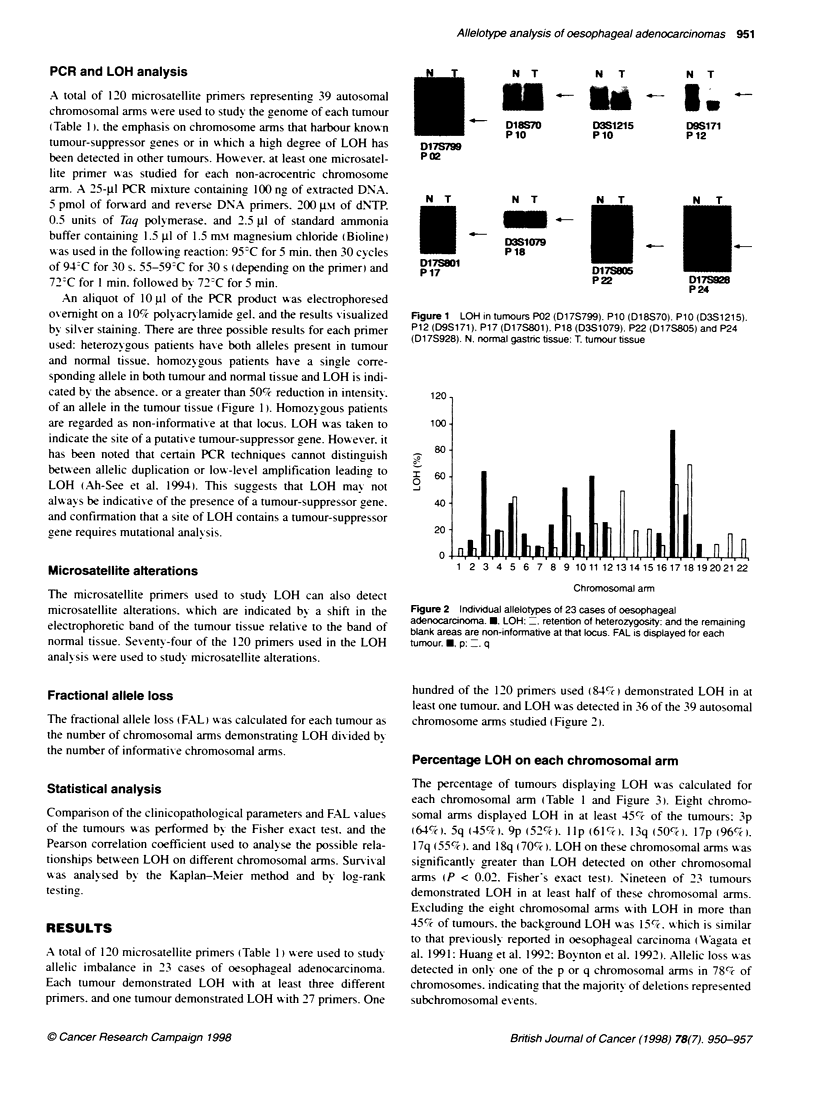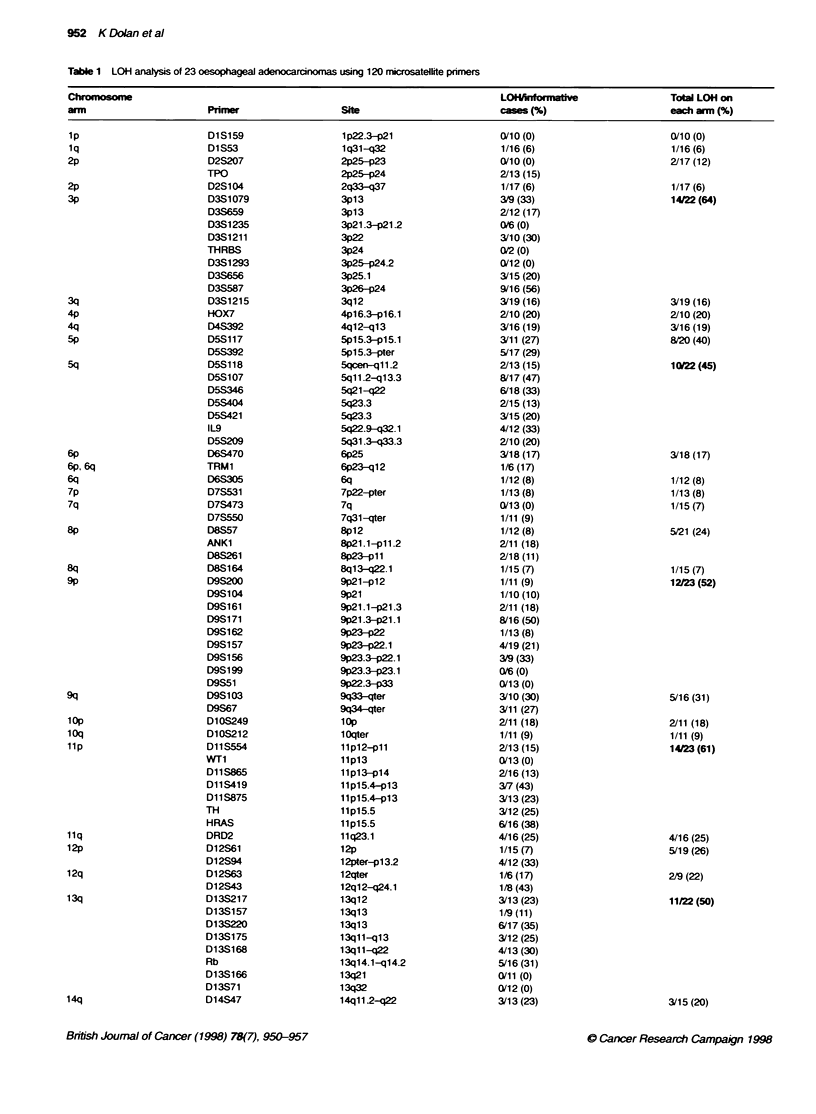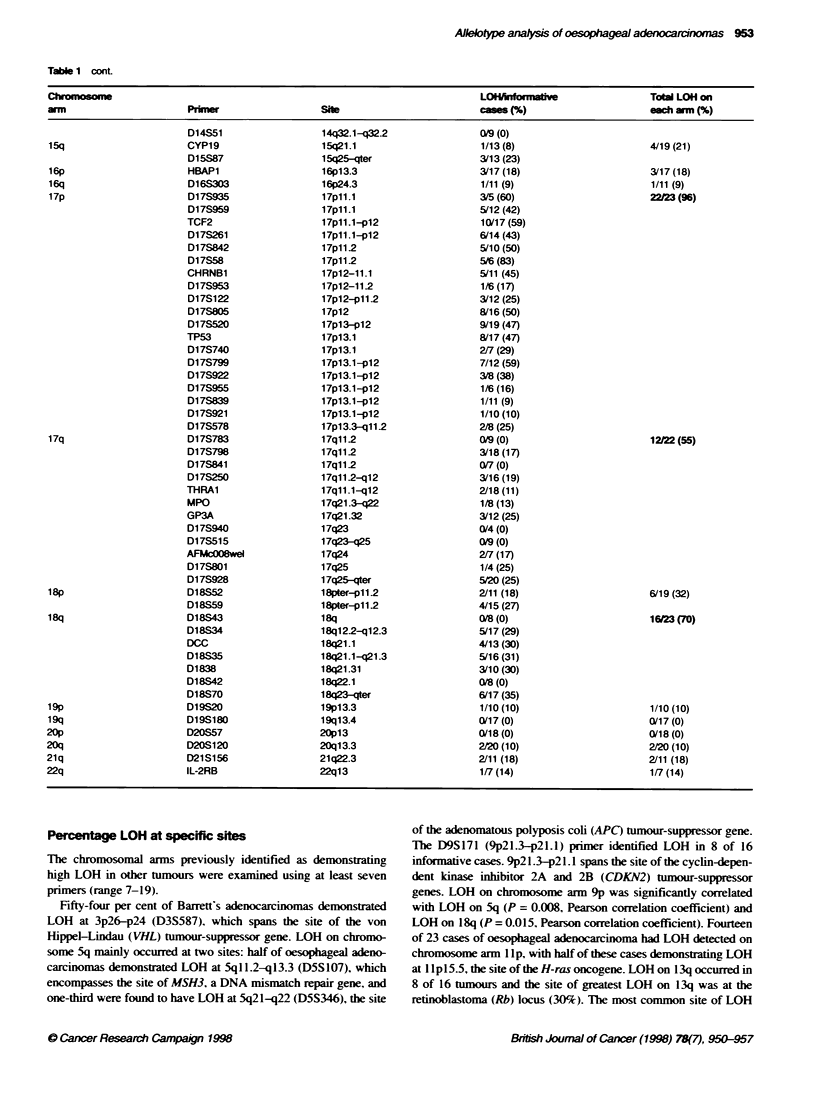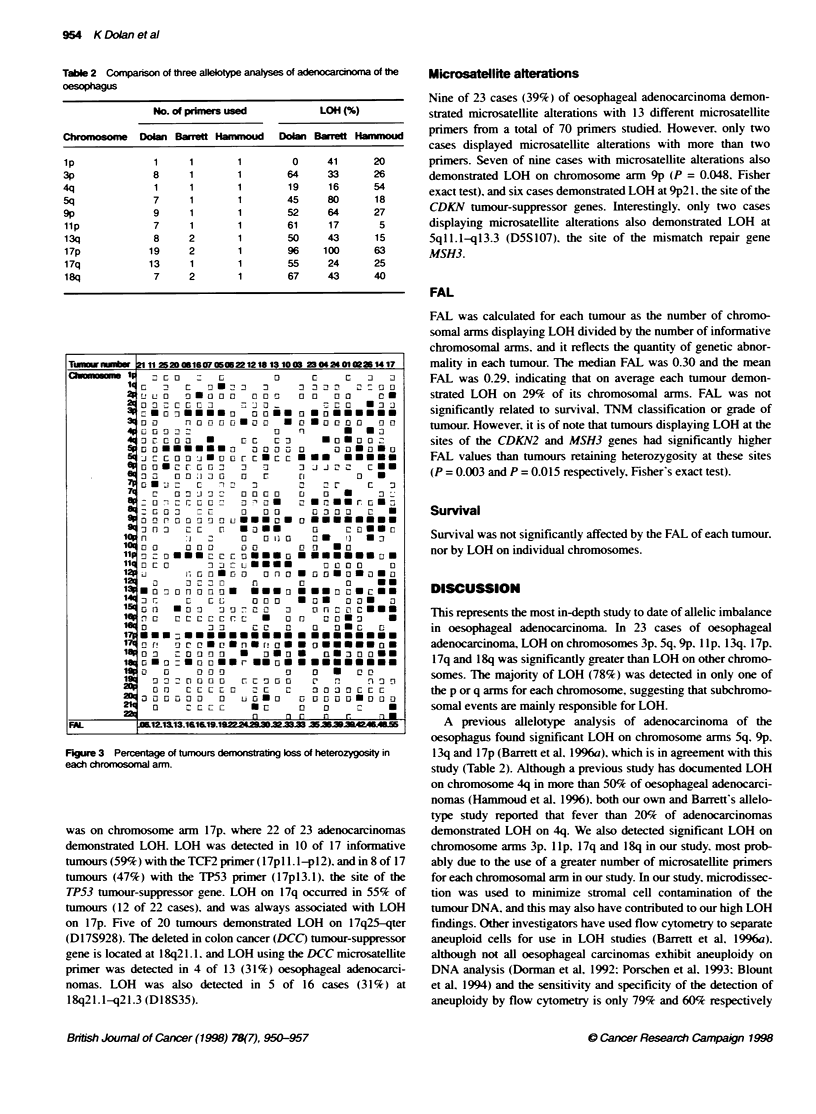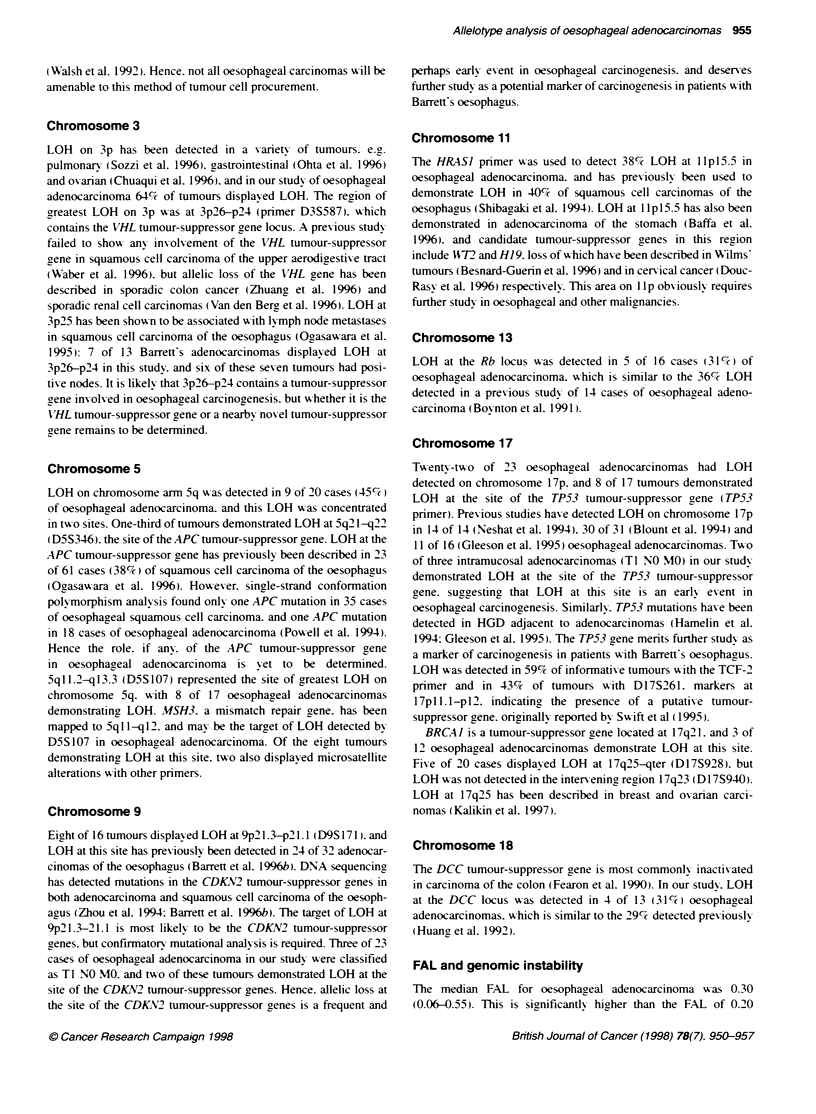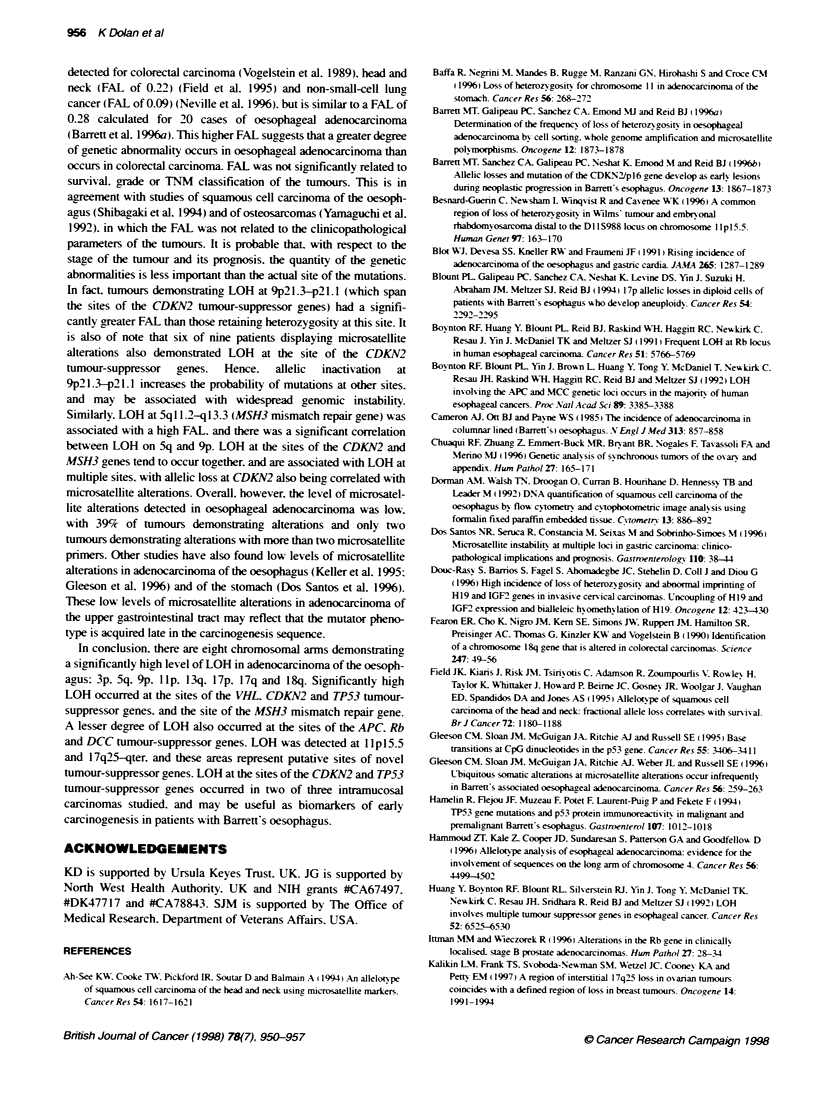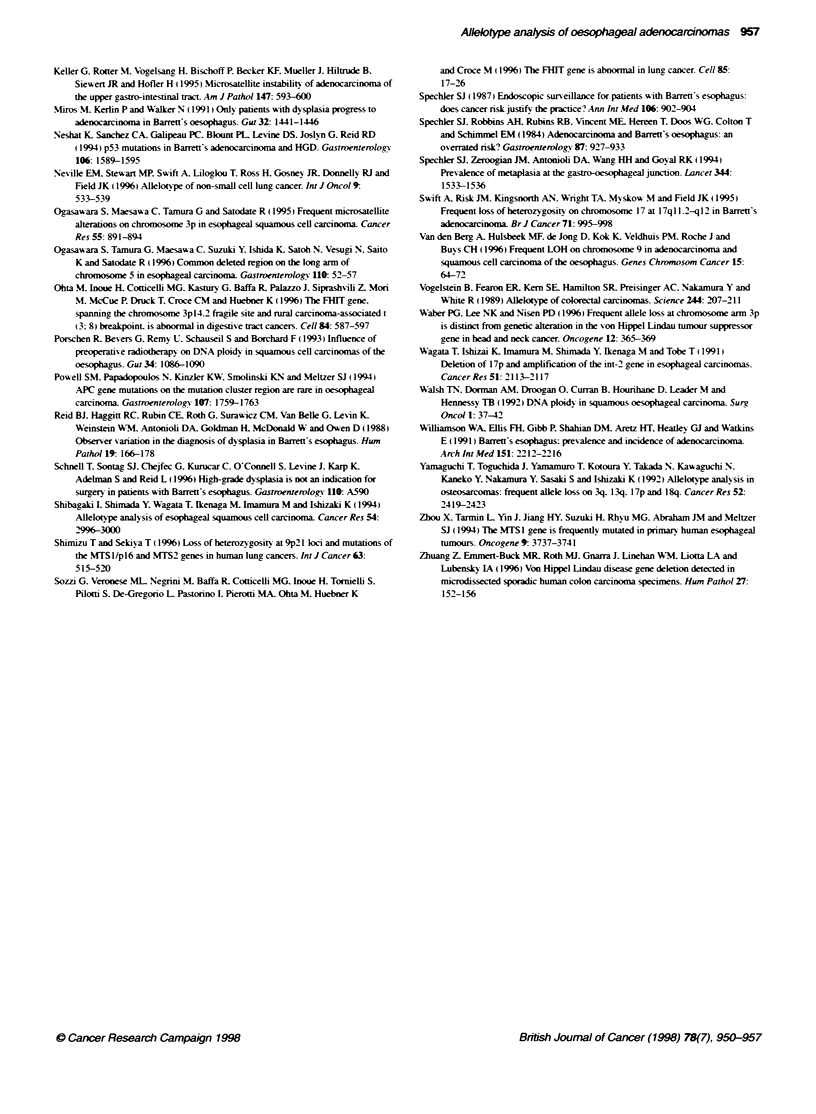# Allelotype analysis of oesophageal adenocarcinoma: loss of heterozygosity occurs at multiple sites.

**DOI:** 10.1038/bjc.1998.607

**Published:** 1998-10

**Authors:** K. Dolan, J. Garde, J. Gosney, M. Sissons, T. Wright, A. N. Kingsnorth, S. J. Walker, R. Sutton, S. J. Meltzer, J. K. Field

**Affiliations:** Molecular Genetics and Oncology Group, Clinical Dental Sciences, Department of Surgery, University of Liverpool, UK.

## Abstract

**Images:**


British Journal of Cancer (1998) 78(7), 950–957

© 1998 Cancer Research Campaign

### Allelotype analysis of oesophageal adenocarcinoma: loss of heterozygosity occurs at multiple sites

**K Dolan[1,2], J Garde[1], J Gosney[3], M Sissons[4], T Wright[2], AN Kingsnorth[2], SJ Walker[5], R Sutton[2], SJ Meltzer[6] and JK Field[1]**

[1]Molecular Genetics and Oncology Group. Clinical Dental Sciences. Departments of [2]Surgery and [3]Pathology. University of Liverpool. Liverpool L69 3BX. UK: Departments of [4]Pathology and [5]Surgery. Blackpool Victoria Infirmary. Blackpool FY3 8NR. UK: [6]Gastroenterology Division. University of Maryland. Baltimore. MD. USA

**Summary** Deletions of tumour-suppressor genes can be detected by loss of heterozygosity (LOH) studies, which were performed on 23 cases of adenocarcinoma of the oesophagus, using 120 microsatellite primers covering all non-acrocentric autosomal chromosome arms. The chromosomal arms most frequently demonstrating LOH were 3p (64% of tumours), 5q (45%), 9p (52%), 11p (61%), 13q (50%), 17p (96%), 17q (55%) and 18q (70%). LOH on 3p, 9p, 13q, 17p and 18q occurred mainly within the loci of the *VHL*, *CDKN2*, *Rb*, *TP53* and *DCC* tumour-suppressor genes respectively. LOH on 5q occurred at the sites of the *MSH3* mismatch repair gene and the *APC* tumour-suppressor gene. 11p15.5 and 17q25–qter represented areas of greatest LOH on chromosomes 11p and 17q, and are putative sites of novel tumour-suppressor genes. LOH on 9p was significantly associated with LOH on 5q, and tumours demonstrating LOH at both the *CDKN2* (9p21) and *MSH3* (5q11–q12) genes had a significantly higher fractional allele loss than those retaining heterozygosity at these sites. Six of nine carcinomas displaying microsatellite alterations also demonstrated LOH at CDKN2, which may be associated with widespread genomic instability. Overall, there are nine sites of LOH associated with oesophageal adenocarcinoma.

**Keywords:** oesophageal adenocarcinoma; loss of heterozygosity; fractional allele loss

The incidence of adenocarcinoma of the oesophagus has increased at a greater rate than any other tumour over the last 20 years (Blot et al. 1991), and is now the most common oesophageal malignancy in certain parts of the Western world (Spechler et al. 1994). The reason for this increase is not clear. However, it is known that Barrett's oesophagus, which occurs in approximately 10% of patients with gastro-oesophageal reflux, is associated with a 30–125 times increased risk of developing adenocarcinoma (Spechler et al. 1984; Cameron et al. 1985; Williamson et al. 1991). It is estimated that approximately 1% of patients with Barrett's oesophagus will develop adenocarcinoma each year, and this can occur up to 20 years after the initial diagnosis of Barrett's oesophagus (Spechler, 1987). The histological progression during this period is considered to follow metaplasia–low-grade dysplasia–high-grade dysplasia–carcinoma (Miros et al. 1991). High-grade dysplasia (HGD) has been used as a marker of future cancer development, but there is interobserver variation in the diagnosis of HGD (Reid et al. 1988) and not all patients with HGD will develop cancer (Schnell et al. 1996).

Attention has therefore focused on molecular biomarkers of carcinogenesis. Loss of function of tumour-suppressor genes resulting from genomic insults has been implicated in the development of several different tumours, and these loss of function mutations may be detected by loss of heterozygosity (LOH) studies (Ittman and Wieczorek, 1996; Shimizu and Sakiya, 1996). LOH studies can lead to the identification of tumour-suppressor genes that are inactivated in the metaplasia–dysplasia–carcinoma progression, and may therefore be useful as biomarkers of future carcinogenesis in patients with Barrett's metaplasia and dysplasia undergoing endoscopic surveillance.

Previous alleleotype analyses have detected LOH in more than 40% of oesophageal adenocarcinomas on chromosome arms 1p, 4q, 5q, 9p, 13q, 17p and 18q (Barrett et al. 1996*a* and Hammoud et al. 1996). These allelotype studies were undertaken with 43 and 39 microsatellite primers respectively. We have performed the most comprehensive genomic study of oesophageal adenocarcinoma to date, covering all of the non-acrocentric chromosome arms with 120 microsatellite primers, enabling identification of putative tumour-suppressor gene sites in oesophageal adenocarcinoma.

### MATERIALS AND METHODS

### Patients

Twenty-three cases of adenocarcinoma of the oesophagus diagnosed between 1992 and 1996 were studied. Twenty of these patients were male and their mean age was 68 years. At present, six of these patients are alive with no signs of recurrent disease.

### DNA extraction

Tissue from the tumour and from normal gastric mucosa were obtained from endoscopic biopsies and from surgical resections, snapped frozen in liquid nitrogen and stored at –70°C. Areas of tumour containing minimal stromal cells were microdissected and DNA extracted from the microdissected tissues using the Nucleon II extraction kit (Scotlab).

*Received 2 December 1997*
*Revised 9 March 1998*
*Accepted 17 March 1998*

*Correspondence to:* JK Field

### PCR and LOH analysis

A total of 120 microsatellite primers representing 39 autosomal chromosomal arms were used to study the genome of each tumour (Table 1), the emphasis on chromosome arms that harbour known tumour-suppressor genes or in which a high degree of LOH has been detected in other tumours. However, at least one microsatellite primer was studied for each non-acrocentric chromosome arm. A 25-µl PCR mixture containing 100 ng of extracted DNA, 5 pmol of forward and reverse DNA primers, 200 µM of dNTP, 0.5 units of *Taq* polymerase, and 2.5 µl of standard ammonia buffer containing 1.5 µl of 1.5 mM magnesium chloride (Bioline) was used in the following reaction: 95°C for 5 min, then 30 cycles of 94°C for 30 s, 55–59°C for 30 s (depending on the primer) and 72°C for 1 min, followed by 72°C for 5 min.

An aliquot of 10 µl of the PCR product was electrophoresed overnight on a 10% polyacrylamide gel, and the results visualized by silver staining. There are three possible results for each primer used: heterozygous patients have both alleles present in tumour and normal tissue, homozygous patients have a single corresponding allele in both tumour and normal tissue and LOH is indicated by the absence, or a greater than 50% reduction in intensity, of an allele in the tumour tissue (Figure 1). Homozygous patients are regarded as non-informative at that locus. LOH was taken to indicate the site of a putative tumour-suppressor gene. However, it has been noted that certain PCR techniques cannot distinguish between allelic duplication or low-level amplification leading to LOH (Ah-See et al, 1994). This suggests that LOH may not always be indicative of the presence of a tumour-suppressor gene, and confirmation that a site of LOH contains a tumour-suppressor gene requires mutational analysis.

### Microsatellite alterations

The microsatellite primers used to study LOH can also detect microsatellite alterations, which are indicated by a shift in the electrophoretic band of the tumour tissue relative to the band of normal tissue. Seventy-four of the 120 primers used in the LOH analysis were used to study microsatellite alterations.

### Fractional allele loss

The fractional allele loss (FAL) was calculated for each tumour as the number of chromosomal arms demonstrating LOH divided by the number of informative chromosomal arms.

### Statistical analysis

Comparison of the clinicopathological parameters and FAL values of the tumours was performed by the Fisher exact test, and the Pearson correlation coefficient used to analyse the possible relationships between LOH on different chromosomal arms. Survival was analysed by the Kaplan–Meier method and by log-rank testing.

### RESULTS

A total of 120 microsatellite primers (Table 1) were used to study allelic imbalance in 23 cases of oesophageal adenocarcinoma. Each tumour demonstrated LOH with at least three different primers, and one tumour demonstrated LOH with 27 primers. One hundred of the 120 primers used (84%) demonstrated LOH in at least one tumour, and LOH was detected in 36 of the 39 autosomal chromosome arms studied (Figure 2).

**Figure 1** LOH in tumours P02 (D17S799), P10 (D18S70), P10 (D3S1215), P12 (D9S171), P17 (D17S801), P18 (D3S1079), P22 (D17S805) and P24 (D17S928). N, normal gastric tissue; T, tumour tissue

**Figure 2** Individual allelotypes of 23 cases of oesophageal adenocarcinoma. ■, LOH; □, retention of heterozygosity; and the remaining blank areas are non-informative at that locus. FAL is displayed for each tumour. ■, p; □, q

### Percentage LOH on each chromosomal arm

The percentage of tumours displaying LOH was calculated for each chromosomal arm (Table 1 and Figure 3). Eight chromosomal arms displayed LOH in at least 45% of the tumours: 3p (64%), 5q (45%), 9p (52%), 11p (61%), 13q (50%), 17p (96%), 17q (55%), and 18q (70%). LOH on these chromosomal arms was significantly greater than LOH detected on other chromosomal arms ($P < 0.02$, Fisher's exact test). Nineteen of 23 tumours demonstrated LOH in at least half of these chromosomal arms. Excluding the eight chromosomal arms with LOH in more than 45% of tumours, the background LOH was 15%, which is similar to that previously reported in oesophageal carcinoma (Wagata et al, 1991; Huang et al, 1992; Boynton et al, 1992). Allelic loss was detected in only one of the p or q chromosomal arms in 78% of chromosomes, indicating that the majority of deletions represented subchromosomal events.

© Cancer Research Campaign 1998

**Table 1** LOH analysis of 23 oesophageal adenocarcinomas using 120 microsatellite primers

| Chromosome arm | Primer | Site | LOH/informative cases (%) | Total LOH on each arm (%) |
|---|---|---|---|---|
| 1p | D1S159 | 1p22.3–p21 | 0/10 (0) | 0/10 (0) |
| 1q | D1S53 | 1q31–q32 | 1/16 (6) | 1/16 (6) |
| 2p | D2S207 | 2p25–p23 | 0/10 (0) | 2/17 (12) |
| | TPO | 2p25–p24 | 2/13 (15) | |
| 2p | D2S104 | 2q33–q37 | 1/17 (6) | 1/17 (6) |
| 3p | D3S1079 | 3p13 | 3/9 (33) | **14/22 (64)** |
| | D3S659 | 3p13 | 2/12 (17) | |
| | D3S1235 | 3p21.3–p21.2 | 0/6 (0) | |
| | D3S1211 | 3p22 | 3/10 (30) | |
| | THRBS | 3p24 | 0/2 (0) | |
| | D3S1293 | 3p25–p24.2 | 0/12 (0) | |
| | D3S656 | 3p25.1 | 3/15 (20) | |
| | D3S587 | 3p26–p24 | 9/16 (56) | |
| 3q | D3S1215 | 3q12 | 3/19 (16) | 3/19 (16) |
| 4p | HOX7 | 4p16.3–p16.1 | 2/10 (20) | 2/10 (20) |
| 4q | D4S392 | 4q12–q13 | 3/16 (19) | 3/16 (19) |
| 5p | D5S117 | 5p15.3–p15.1 | 3/11 (27) | 8/20 (40) |
| | D5S392 | 5p15.3–pter | 5/17 (29) | |
| 5q | D5S118 | 5qcen–q11.2 | 2/13 (15) | **10/22 (45)** |
| | D5S107 | 5q11.2–q13.3 | 8/17 (47) | |
| | D5S346 | 5q21–q22 | 6/18 (33) | |
| | D5S404 | 5q23.3 | 2/15 (13) | |
| | D5S421 | 5q23.3 | 3/15 (20) | |
| | IL9 | 5q22.9–q32.1 | 4/12 (33) | |
| | D5S209 | 5q31.3–q33.3 | 2/10 (20) | |
| 6p | D6S470 | 6p25 | 3/18 (17) | 3/18 (17) |
| 6p, 6q | TRM1 | 6p23–q12 | 1/6 (17) | |
| 6q | D6S305 | 6q | 1/12 (8) | 1/12 (8) |
| 7p | D7S531 | 7p22–pter | 1/13 (8) | 1/13 (8) |
| 7q | D7S473 | 7q | 0/13 (0) | 1/15 (7) |
| | D7S550 | 7q31–qter | 1/11 (9) | |
| 8p | D8S57 | 8p12 | 1/12 (8) | 5/21 (24) |
| | ANK1 | 8p21.1–p11.2 | 2/11 (18) | |
| | D8S261 | 8p23–p11 | 2/18 (11) | |
| 8q | D8S164 | 8q13–q22.1 | 1/15 (7) | 1/15 (7) |
| 9p | D9S200 | 9p21–p12 | 1/11 (9) | **12/23 (52)** |
| | D9S104 | 9p21 | 1/10 (10) | |
| | D9S161 | 9p21.1–p21.3 | 2/11 (18) | |
| | D9S171 | 9p21.3–p21.1 | 8/16 (50) | |
| | D9S162 | 9p23–p22 | 1/13 (8) | |
| | D9S157 | 9p23–p22.1 | 4/19 (21) | |
| | D9S156 | 9p23.3–p22.1 | 3/9 (33) | |
| | D9S199 | 9p23.3–p23.1 | 0/6 (0) | |
| | D9S51 | 9p22.3–p33 | 0/13 (0) | |
| 9q | D9S103 | 9q33–qter | 3/10 (30) | 5/16 (31) |
| | D9S67 | 9q34–qter | 3/11 (27) | |
| 10p | D10S249 | 10p | 2/11 (18) | 2/11 (18) |
| 10q | D10S212 | 10qter | 1/11 (9) | 1/11 (9) |
| 11p | D11S554 | 11p12–p11 | 2/13 (15) | **14/23 (61)** |
| | WT1 | 11p13 | 0/13 (0) | |
| | D11S865 | 11p13–p14 | 2/16 (13) | |
| | D11S419 | 11p15.4–p13 | 3/7 (43) | |
| | D11S875 | 11p15.4–p13 | 3/13 (23) | |
| | TH | 11p15.5 | 3/12 (25) | |
| | HRAS | 11p15.5 | 6/16 (38) | |
| 11q | DRD2 | 11q23.1 | 4/16 (25) | 4/16 (25) |
| 12p | D12S61 | 12p | 1/15 (7) | 5/19 (26) |
| | D12S94 | 12pter–p13.2 | 4/12 (33) | |
| 12q | D12S63 | 12qter | 1/6 (17) | 2/9 (22) |
| | D12S43 | 12q12–q24.1 | 1/8 (43) | |
| 13q | D13S217 | 13q12 | 3/13 (23) | **11/22 (50)** |
| | D13S157 | 13q13 | 1/9 (11) | |
| | D13S220 | 13q13 | 6/17 (35) | |
| | D13S175 | 13q11–q13 | 3/12 (25) | |
| | D13S168 | 13q11–q22 | 4/13 (30) | |
| | Rb | 13q14.1–q14.2 | 5/16 (31) | |
| | D13S166 | 13q21 | 0/11 (0) | |
| | D13S71 | 13q32 | 0/12 (0) | |
| 14q | D14S47 | 14q11.2–q22 | 3/13 (23) | 3/15 (20) |

© Cancer Research Campaign 1998

**Table 1** cont.

| Chromosome arm | Primer | Site | LOH/informative cases (%) | Total LOH on each arm (%) |
|---|---|---|---|---|
| | D14S51 | 14q32.1–q32.2 | 0/9 (0) | |
| 15q | CYP19 | 15q21.1 | 1/13 (8) | 4/19 (21) |
| | D15S87 | 15q25–qter | 3/13 (23) | |
| 16p | HBAP1 | 16p13.3 | 3/17 (18) | 3/17 (18) |
| 16q | D16S303 | 16p24.3 | 1/11 (9) | 1/11 (9) |
| 17p | D17S935 | 17p11.1 | 3/5 (60) | **22/23 (96)** |
| | D17S959 | 17p11.1 | 5/12 (42) | |
| | TCF2 | 17p11.1–p12 | 10/17 (59) | |
| | D17S261 | 17p11.1–p12 | 6/14 (43) | |
| | D17S842 | 17p11.2 | 5/10 (50) | |
| | D17S58 | 17p11.2 | 5/6 (83) | |
| | CHRNB1 | 17p12–11.1 | 5/11 (45) | |
| | D17S953 | 17p12–11.2 | 1/6 (17) | |
| | D17S122 | 17p12–p11.2 | 3/12 (25) | |
| | D17S805 | 17p12 | 8/16 (50) | |
| | D17S520 | 17p13–p12 | 9/19 (47) | |
| | TP53 | 17p13.1 | 8/17 (47) | |
| | D17S740 | 17p13.1 | 2/7 (29) | |
| | D17S799 | 17p13.1–p12 | 7/12 (59) | |
| | D17S922 | 17p13.1–p12 | 3/8 (38) | |
| | D17S955 | 17p13.1–p12 | 1/6 (16) | |
| | D17S839 | 17p13.1–p12 | 1/11 (9) | |
| | D17S921 | 17p13.1–p12 | 1/10 (10) | |
| | D17S578 | 17p13.3–q11.2 | 2/8 (25) | |
| 17q | D17S783 | 17q11.2 | 0/9 (0) | **12/22 (55)** |
| | D17S798 | 17q11.2 | 3/18 (17) | |
| | D17S841 | 17q11.2 | 0/7 (0) | |
| | D17S250 | 17q11.2–q12 | 3/16 (19) | |
| | THRA1 | 17q11.1–q12 | 2/18 (11) | |
| | MPO | 17q21.3–q22 | 1/8 (13) | |
| | GP3A | 17q21.32 | 3/12 (25) | |
| | D17S940 | 17q23 | 0/4 (0) | |
| | D17S515 | 17q23–q25 | 0/9 (0) | |
| | AFMc008wel | 17q24 | 2/7 (17) | |
| | D17S801 | 17q25 | 1/4 (25) | |
| | D17S928 | 17q25–qter | 5/20 (25) | |
| 18p | D18S52 | 18pter–p11.2 | 2/11 (18) | 6/19 (32) |
| | D18S59 | 18pter–p11.2 | 4/15 (27) | |
| 18q | D18S43 | 18q | 0/8 (0) | **16/23 (70)** |
| | D18S34 | 18q12.2–q12.3 | 5/17 (29) | |
| | DCC | 18q21.1 | 4/13 (30) | |
| | D18S35 | 18q21.1–q21.3 | 5/16 (31) | |
| | D1838 | 18q21.31 | 3/10 (30) | |
| | D18S42 | 18q22.1 | 0/8 (0) | |
| | D18S70 | 18q23–qter | 6/17 (35) | |
| 19p | D19S20 | 19p13.3 | 1/10 (10) | 1/10 (10) |
| 19q | D19S180 | 19q13.4 | 0/17 (0) | 0/17 (0) |
| 20p | D20S57 | 20p13 | 0/18 (0) | 0/18 (0) |
| 20q | D20S120 | 20q13.3 | 2/20 (10) | 2/20 (10) |
| 21q | D21S156 | 21q22.3 | 2/11 (18) | 2/11 (18) |
| 22q | IL-2RB | 22q13 | 1/7 (14) | 1/7 (14) |

### Percentage LOH at specific sites

The chromosomal arms previously identified as demonstrating high LOH in other tumours were examined using at least seven primers (range 7–19).

Fifty-four per cent of Barrett's adenocarcinomas demonstrated LOH at 3p26–p24 (D3S587), which spans the site of the von Hippel–Lindau (*VHL*) tumour-suppressor gene. LOH on chromosome 5q mainly occurred at two sites: half of oesophageal adenocarcinomas demonstrated LOH at 5q11.2–q13.3 (D5S107), which encompasses the site of *MSH3*, a DNA mismatch repair gene, and one-third were found to have LOH at 5q21–q22 (D5S346), the site of the adenomatous polyposis coli (*APC*) tumour-suppressor gene. The D9S171 (9p21.3–p21.1) primer identified LOH in 8 of 16 informative cases. 9p21.3–p21.1 spans the site of the cyclin-dependent kinase inhibitor 2A and 2B (*CDKN2*) tumour-suppressor genes. LOH on chromosome arm 9p was significantly correlated with LOH on 5q ($P = 0.008$, Pearson correlation coefficient) and LOH on 18q ($P = 0.015$, Pearson correlation coefficient). Fourteen of 23 cases of oesophageal adenocarcinoma had LOH detected on chromosome arm 11p, with half of these cases demonstrating LOH at 11p15.5, the site of the *H-ras* oncogene. LOH on 13q occurred in 8 of 16 tumours and the site of greatest LOH on 13q was at the retinoblastoma (*Rb*) locus (30%). The most common site of LOH

**Table 2** Comparison of three allelotype analyses of adenocarcinoma of the oesophagus

| | No. of primers used | | | LOH (%) | | |
|---|---|---|---|---|---|---|
| Chromosome | Dolan | Barrett | Hammoud | Dolan | Barrett | Hammoud |
| 1p | 1 | 1 | 1 | 0 | 41 | 20 |
| 3p | 8 | 1 | 1 | 64 | 33 | 26 |
| 4q | 1 | 1 | 1 | 19 | 16 | 54 |
| 5q | 7 | 1 | 1 | 45 | 80 | 18 |
| 9p | 9 | 1 | 1 | 52 | 64 | 27 |
| 11p | 7 | 1 | 1 | 61 | 17 | 5 |
| 13q | 8 | 2 | 1 | 50 | 43 | 15 |
| 17p | 19 | 2 | 1 | 96 | 100 | 63 |
| 17q | 13 | 1 | 1 | 55 | 24 | 25 |
| 18q | 7 | 2 | 1 | 67 | 43 | 40 |

**Figure 3** Percentage of tumours demonstrating loss of heterozygosity in each chromosomal arm.

was on chromosome arm 17p, where 22 of 23 adenocarcinomas demonstrated LOH. LOH was detected in 10 of 17 informative tumours (59%) with the TCF2 primer (17p11.1–p12), and in 8 of 17 tumours (47%) with the TP53 primer (17p13.1), the site of the *TP53* tumour-suppressor gene. LOH on 17q occurred in 55% of tumours (12 of 22 cases), and was always associated with LOH on 17p. Five of 20 tumours demonstrated LOH on 17q25–qter (D17S928). The deleted in colon cancer (*DCC*) tumour-suppressor gene is located at 18q21.1, and LOH using the *DCC* microsatellite primer was detected in 4 of 13 (31%) oesophageal adenocarcinomas. LOH was also detected in 5 of 16 cases (31%) at 18q21.1–q21.3 (D18S35).

### Microsatellite alterations

Nine of 23 cases (39%) of oesophageal adenocarcinoma demonstrated microsatellite alterations with 13 different microsatellite primers from a total of 70 primers studied. However, only two cases displayed microsatellite alterations with more than two primers. Seven of nine cases with microsatellite alterations also demonstrated LOH on chromosome arm 9p ($P$ = 0.048, Fisher exact test), and six cases demonstrated LOH at 9p21, the site of the *CDKN* tumour-suppressor genes. Interestingly, only two cases displaying microsatellite alterations also demonstrated LOH at 5q11.1–q13.3 (D5S107), the site of the mismatch repair gene *MSH3*.

### FAL

FAL was calculated for each tumour as the number of chromosomal arms displaying LOH divided by the number of informative chromosomal arms, and it reflects the quantity of genetic abnormality in each tumour. The median FAL was 0.30 and the mean FAL was 0.29, indicating that on average each tumour demonstrated LOH on 29% of its chromosomal arms. FAL was not significantly related to survival, TNM classification or grade of tumour. However, it is of note that tumours displaying LOH at the sites of the *CDKN2* and *MSH3* genes had significantly higher FAL values than tumours retaining heterozygosity at these sites ($P = 0.003$ and $P = 0.015$ respectively, Fisher's exact test).

### Survival

Survival was not significantly affected by the FAL of each tumour, nor by LOH on individual chromosomes.

### DISCUSSION

This represents the most in-depth study to date of allelic imbalance in oesophageal adenocarcinoma. In 23 cases of oesophageal adenocarcinoma, LOH on chromosomes 3p, 5q, 9p, 11p, 13q, 17p, 17q and 18q was significantly greater than LOH on other chromosomes. The majority of LOH (78%) was detected in only one of the p or q arms for each chromosome, suggesting that subchromosomal events are mainly responsible for LOH.

A previous allelotype analysis of adenocarcinoma of the oesophagus found significant LOH on chromosome arms 5q, 9p, 13q and 17p (Barrett et al, 1996*a*), which is in agreement with this study (Table 2). Although a previous study has documented LOH on chromosome 4q in more than 50% of oesophageal adenocarcinomas (Hammoud et al, 1996), both our own and Barrett's allelotype study reported that fever than 20% of adenocarcinomas demonstrated LOH on 4q. We also detected significant LOH on chromosome arms 3p, 11p, 17q and 18q in our study, most probably due to the use of a greater number of microsatellite primers for each chromosomal arm in our study. In our study, microdissection was used to minimize stromal cell contamination of the tumour DNA, and this may also have contributed to our high LOH findings. Other investigators have used flow cytometry to separate aneuploid cells for use in LOH studies (Barrett et al, 1996*a*), although not all oesophageal carcinomas exhibit aneuploidy on DNA analysis (Dorman et al, 1992; Porschen et al, 1993; Blount et al, 1994) and the sensitivity and specificity of the detection of aneuploidy by flow cytometry is only 79% and 60% respectively

© Cancer Research Campaign 1998

(Walsh et al. 1992). Hence, not all oesophageal carcinomas will be amenable to this method of tumour cell procurement.

### Chromosome 3

LOH on 3p has been detected in a variety of tumours, e.g. pulmonary (Sozzi et al. 1996), gastrointestinal (Ohta et al. 1996) and ovarian (Chuaqui et al. 1996), and in our study of oesophageal adenocarcinoma 64% of tumours displayed LOH. The region of greatest LOH on 3p was at 3p26–p24 (primer D3S587), which contains the *VHL* tumour-suppressor gene locus. A previous study failed to show any involvement of the *VHL* tumour-suppressor gene in squamous cell carcinoma of the upper aerodigestive tract (Waber et al. 1996), but allelic loss of the *VHL* gene has been described in sporadic colon cancer (Zhuang et al. 1996) and sporadic renal cell carcinomas (Van den Berg et al. 1996). LOH at 3p25 has been shown to be associated with lymph node metastases in squamous cell carcinoma of the oesophagus (Ogasawara et al. 1995): 7 of 13 Barrett's adenocarcinomas displayed LOH at 3p26–p24 in this study, and six of these seven tumours had positive nodes. It is likely that 3p26–p24 contains a tumour-suppressor gene involved in oesophageal carcinogenesis, but whether it is the *VHL* tumour-suppressor gene or a nearby novel tumour-suppressor gene remains to be determined.

### Chromosome 5

LOH on chromosome arm 5q was detected in 9 of 20 cases (45%) of oesophageal adenocarcinoma, and this LOH was concentrated in two sites. One-third of tumours demonstrated LOH at 5q21–q22 (D5S346), the site of the *APC* tumour-suppressor gene. LOH at the *APC* tumour-suppressor gene has previously been described in 23 of 61 cases (38%) of squamous cell carcinoma of the oesophagus (Ogasawara et al. 1996). However, single-strand conformation polymorphism analysis found only one *APC* mutation in 35 cases of oesophageal squamous cell carcinoma, and one *APC* mutation in 18 cases of oesophageal adenocarcinoma (Powell et al. 1994). Hence the role, if any, of the *APC* tumour-suppressor gene in oesophageal adenocarcinoma is yet to be determined. 5q11.2–q13.3 (D5S107) represented the site of greatest LOH on chromosome 5q, with 8 of 17 oesophageal adenocarcinomas demonstrating LOH. *MSH3*, a mismatch repair gene, has been mapped to 5q11–q12, and may be the target of LOH detected by D5S107 in oesophageal adenocarcinoma. Of the eight tumours demonstrating LOH at this site, two also displayed microsatellite alterations with other primers.

### Chromosome 9

Eight of 16 tumours displayed LOH at 9p21.3–p21.1 (D9S171), and LOH at this site has previously been detected in 24 of 32 adenocarcinomas of the oesophagus (Barrett et al. 1996*b*). DNA sequencing has detected mutations in the *CDKN2* tumour-suppressor genes in both adenocarcinoma and squamous cell carcinoma of the oesophagus (Zhou et al. 1994; Barrett et al. 1996*b*). The target of LOH at 9p21.3–21.1 is most likely to be the *CDKN2* tumour-suppressor genes, but confirmatory mutational analysis is required. Three of 23 cases of oesophageal adenocarcinoma in our study were classified as T1 N0 M0, and two of these tumours demonstrated LOH at the site of the *CDKN2* tumour-suppressor genes. Hence, allelic loss at the site of the *CDKN2* tumour-suppressor genes is a frequent and perhaps early event in oesophageal carcinogenesis, and deserves further study as a potential marker of carcinogenesis in patients with Barrett's oesophagus.

### Chromosome 11

The *HRAS1* primer was used to detect 38% LOH at 11p15.5 in oesophageal adenocarcinoma, and has previously been used to demonstrate LOH in 40% of squamous cell carcinomas of the oesophagus (Shibagaki et al. 1994). LOH at 11p15.5 has also been demonstrated in adenocarcinoma of the stomach (Baffa et al. 1996), and candidate tumour-suppressor genes in this region include *WT2* and *H19*, loss of which have been described in Wilms' tumours (Besnard-Guerin et al. 1996) and in cervical cancer (Douc-Rasy et al. 1996) respectively. This area on 11p obviously requires further study in oesophageal and other malignancies.

### Chromosome 13

LOH at the *Rb* locus was detected in 5 of 16 cases (31%) of oesophageal adenocarcinoma, which is similar to the 36% LOH detected in a previous study of 14 cases of oesophageal adenocarcinoma (Boynton et al. 1991).

### Chromosome 17

Twenty-two of 23 oesophageal adenocarcinomas had LOH detected on chromosome 17p, and 8 of 17 tumours demonstrated LOH at the site of the *TP53* tumour-suppressor gene (*TP53* primer). Previous studies have detected LOH on chromosome 17p in 14 of 14 (Neshat et al. 1994), 30 of 31 (Blount et al. 1994) and 11 of 16 (Gleeson et al. 1995) oesophageal adenocarcinomas. Two of three intramucosal adenocarcinomas (T1 N0 M0) in our study demonstrated LOH at the site of the *TP53* tumour-suppressor gene, suggesting that LOH at this site is an early event in oesophageal carcinogenesis. Similarly, *TP53* mutations have been detected in HGD adjacent to adenocarcinomas (Hamelin et al. 1994; Gleeson et al. 1995). The *TP53* gene merits further study as a marker of carcinogenesis in patients with Barrett's oesophagus. LOH was detected in 59% of informative tumours with the TCF-2 primer and in 43% of tumours with D17S261, markers at 17p11.1–p12, indicating the presence of a putative tumour-suppressor gene, originally reported by Swift et al (1995).

*BRCA1* is a tumour-suppressor gene located at 17q21, and 3 of 12 oesophageal adenocarcinomas demonstrate LOH at this site. Five of 20 cases displayed LOH at 17q25–qter (D17S928), but LOH was not detected in the intervening region 17q23 (D17S940). LOH at 17q25 has been described in breast and ovarian carcinomas (Kalikin et al. 1997).

### Chromosome 18

The *DCC* tumour-suppressor gene is most commonly inactivated in carcinoma of the colon (Fearon et al. 1990). In our study, LOH at the *DCC* locus was detected in 4 of 13 (31%) oesophageal adenocarcinomas, which is similar to the 29% detected previously (Huang et al. 1992).

### FAL and genomic instability

The median FAL for oesophageal adenocarcinoma was 0.30 (0.06–0.55). This is significantly higher than the FAL of 0.20

© Cancer Research Campaign 1998 

detected for colorectal carcinoma (Vogelstein et al. 1989), head and neck (FAL of 0.22) (Field et al. 1995) and non-small-cell lung cancer (FAL of 0.09) (Neville et al. 1996), but is similar to a FAL of 0.28 calculated for 20 cases of oesophageal adenocarcinoma (Barrett et al. 1996*a*). This higher FAL suggests that a greater degree of genetic abnormality occurs in oesophageal adenocarcinoma than occurs in colorectal carcinoma. FAL was not significantly related to survival, grade or TNM classification of the tumours. This is in agreement with studies of squamous cell carcinoma of the oesophagus (Shibagaki et al. 1994) and of osteosarcomas (Yamaguchi et al. 1992), in which the FAL was not related to the clinicopathological parameters of the tumours. It is probable that, with respect to the stage of the tumour and its prognosis, the quantity of the genetic abnormalities is less important than the actual site of the mutations. In fact, tumours demonstrating LOH at 9p21.3–p21.1 (which span the sites of the *CDKN2* tumour-suppressor genes) had a significantly greater FAL than those retaining heterozygosity at this site. It is also of note that six of nine patients displaying microsatellite alterations also demonstrated LOH at the site of the *CDKN2* tumour-suppressor genes. Hence, allelic inactivation at 9p21.3–p21.1 increases the probability of mutations at other sites, and may be associated with widespread genomic instability. Similarly, LOH at 5q11.2–q13.3 (*MSH3* mismatch repair gene) was associated with a high FAL, and there was a significant correlation between LOH on 5q and 9p. LOH at the sites of the *CDKN2* and *MSH3* genes tend to occur together, and are associated with LOH at multiple sites, with allelic loss at *CDKN2* also being correlated with microsatellite alterations. Overall, however, the level of microsatellite alterations detected in oesophageal adenocarcinoma was low, with 39% of tumours demonstrating alterations and only two tumours demonstrating alterations with more than two microsatellite primers. Other studies have also found low levels of microsatellite alterations in adenocarcinoma of the oesophagus (Keller et al. 1995; Gleeson et al. 1996) and of the stomach (Dos Santos et al. 1996). These low levels of microsatellite alterations in adenocarcinoma of the upper gastrointestinal tract may reflect that the mutator phenotype is acquired late in the carcinogenesis sequence.

In conclusion, there are eight chromosomal arms demonstrating a significantly high level of LOH in adenocarcinoma of the oesophagus; 3p, 5q, 9p, 11p, 13q, 17p, 17q and 18q. Significantly high LOH occurred at the sites of the *VHL*, *CDKN2* and *TP53* tumour-suppressor genes, and the site of the *MSH3* mismatch repair gene. A lesser degree of LOH also occurred at the sites of the *APC*, *Rb* and *DCC* tumour-suppressor genes. LOH was detected at 11p15.5 and 17q25–qter, and these areas represent putative sites of novel tumour-suppressor genes. LOH at the sites of the *CDKN2* and *TP53* tumour-suppressor genes occurred in two of three intramucosal carcinomas studied, and may be useful as biomarkers of early carcinogenesis in patients with Barrett's oesophagus.

### ACKNOWLEDGEMENTS

KD is supported by Ursula Keyes Trust, UK. JG is supported by North West Health Authority, UK and NIH grants #CA67497, #DK47717 and #CA78843. SJM is supported by The Office of Medical Research, Department of Veterans Affairs, USA.

### REFERENCES

Ah-See KW, Cooke TW, Pickford IR, Soutar D and Balmain A (1994) An allelotype of squamous cell carcinoma of the head and neck using microsatellite markers. *Cancer Res* **54**: 1617–1621

Baffa R, Negrini M, Mandes B, Rugge M, Ranzani GN, Hirohashi S and Croce CM (1996) Loss of heterozygosity for chromosome 11 in adenocarcinoma of the stomach. *Cancer Res* **56**: 268–272

Barrett MT, Galipeau PC, Sanchez CA, Emond MJ and Reid BJ (1996*a*) Determination of the frequency of loss of heterozygosity in oesophageal adenocarcinoma by cell sorting, whole genome amplification and microsatellite polymorphisms. *Oncogene* **12**: 1873–1878

Barrett MT, Sanchez CA, Galipeau PC, Neshat K, Emond M and Reid BJ (1996*b*) Allelic losses and mutation of the CDKN2/p16 gene develop as early lesions during neoplastic progression in Barrett's esophagus. *Oncogene* **13**: 1867–1873

Besnard-Guerin C, Newsham I, Winqvist R and Cavenee WK (1996) A common region of loss of heterozygosity in Wilms' tumour and embryonal rhabdomyosarcoma distal to the D11S988 locus on chromosome 11p15.5. *Human Genet* **97**: 163–170

Blot WJ, Devesa SS, Kneller RW and Fraumeni JF (1991) Rising incidence of adenocarcinoma of the oesophagus and gastric cardia. *JAMA* **265**: 1287–1289

Blount PL, Galipeau PC, Sanchez CA, Neshat K, Levine DS, Yin J, Suzuki H, Abraham JM, Meltzer SJ, Reid BJ (1994) 17p allelic losses in diploid cells of patients with Barrett's esophagus who develop aneuploidy. *Cancer Res* **54**: 2292–2295

Boynton RF, Huang Y, Blount PL, Reid BJ, Raskind WH, Haggitt RC, Newkirk C, Resau J, Yin J, McDaniel TK and Meltzer SJ (1991) Frequent LOH at Rb locus in human esophageal carcinoma. *Cancer Res* **51**: 5766–5769

Boynton RF, Blount PL, Yin J, Brown L, Huang Y, Tong Y, McDaniel T, Newkirk C, Resau JH, Raskind WH, Haggitt RC, Reid BJ and Meltzer SJ (1992) LOH involving the APC and MCC genetic loci occurs in the majority of human esophageal cancers. *Proc Natl Acad Sci* **89**: 3385–3388

Cameron AJ, Ott BJ and Payne WS (1985) The incidence of adenocarcinoma in columnar lined (Barrett's) oesophagus. *N Engl J Med* **313**: 857–858

Chuaqui RF, Zhuang Z, Emmert-Buck MR, Bryant BR, Nogales F, Tavassoli FA and Merino MJ (1996) Genetic analysis of synchronous tumors of the ovary and appendix. *Hum Pathol* **27**: 165–171

Dorman AM, Walsh TN, Droogan O, Curran B, Hourihane D, Hennessy TB and Leader M (1992) DNA quantification of squamous cell carcinoma of the oesophagus by flow cytometry and cytophotometric image analysis using formalin fixed paraffin embedded tissue. *Cytometry* **13**: 886–892

Dos Santos NR, Seruca R, Constancia M, Seixas M and Sobrinho-Simoes M (1996) Microsatellite instability at multiple loci in gastric carcinoma: clinicopathological implications and prognosis. *Gastroenterology* **110**: 38–44

Douc-Rasy S, Barrios S, Fagel S, Ahomadegbe JC, Stehelin D, Coll J and Diou G (1996) High incidence of loss of heterozygosity and abnormal imprinting of H19 and IGF2 genes in invasive cervical carcinomas. Uncoupling of H19 and IGF2 expression and bialleleic hyomethylation of H19. *Oncogene* **12**: 423–430

Fearon ER, Cho K, Nigro JM, Kern SE, Simons JW, Ruppert JM, Hamilton SR, Preisinger AC, Thomas G, Kinzler KW and Vogelstein B (1990) Identification of a chromosome 18q gene that is altered in colorectal carcinomas. *Science* **247**: 49–56

Field JK, Kiaris J, Risk JM, Tsiriyotis C, Adamson R, Zoumpourlis V, Rowley H, Taylor K, Whittaker J, Howard P, Beirne JC, Gosney JR, Woolgar J, Vaughan ED, Spandidos DA and Jones AS (1995) Allelotype of squamous cell carcinoma of the head and neck: fractional allele loss correlates with survival. *Br J Cancer* **72**: 1180–1188

Gleeson CM, Sloan JM, McGuigan JA, Ritchie AJ and Russell SE (1995) Base transitions at CpG dinucleotides in the p53 gene. *Cancer Res* **55**: 3406–3411

Gleeson CM, Sloan JM, McGuigan JA, Ritchie AJ, Weber JL and Russell SE (1996) Ubiquitous somatic alterations at microsatellite alterations occur infrequently in Barrett's associated oesophageal adenocarcinoma. *Cancer Res* **56**: 259–263

Hamelin R, Flejou JF, Muzeau F, Potet F, Laurent-Puig P and Fekete F (1994) TP53 gene mutations and p53 protein immunoreactivity in malignant and premalignant Barrett's esophagus. *Gastroenterol* **107**: 1012–1018

Hammoud ZT, Kale Z, Cooper JD, Sundaresan S, Patterson GA and Goodfellow D (1996) Allelotype analysis of esophageal adenocarcinoma: evidence for the involvement of sequences on the long arm of chromosome 4. *Cancer Res* **56**: 4499–4502

Huang Y, Boynton RF, Blount RL, Silverstein RJ, Yin J, Tong Y, McDaniel TK, Newkirk C, Resau JH, Sridhara R, Reid BJ and Meltzer SJ (1992) LOH involves multiple tumour suppressor genes in esophageal cancer. *Cancer Res* **52**: 6525–6530

Ittman MM and Wieczorek R (1996) Alterations in the Rb gene in clinically localised, stage B prostate adenocarcinomas. *Hum Pathol* **27**: 28–34

Kalikin LM, Frank TS, Svoboda-Newman SM, Wetzel JC, Cooney KA and Petty EM (1997) A region of interstitial 17q25 loss in ovarian tumours coincides with a defined region of loss in breast tumours. *Oncogene* **14**: 1991–1994

© Cancer Research Campaign 1998

Keller G, Rotter M, Vogelsang H, Bischoff P, Becker KF, Mueller J, Hiltrude B, Siewert JR and Hofler H (1995) Microsatellite instability of adenocarcinoma of the upper gastro-intestinal tract. *Am J Pathol* **147**: 593–600

Miros M, Kerlin P and Walker N (1991) Only patients with dysplasia progress to adenocarcinoma in Barrett's oesophagus. *Gut* **32**: 1441–1446

Neshat K, Sanchez CA, Galipeau PC, Blount PL, Levine DS, Joslyn G, Reid RD (1994) p53 mutations in Barrett's adenocarcinoma and HGD. *Gastroenterology* **106**: 1589–1595

Neville EM, Stewart MP, Swift A, Liloglou T, Ross H, Gosney JR, Donnelly RJ and Field JK (1996) Allelotype of non-small cell lung cancer. *Int J Oncol* **9**: 533–539

Ogasawara S, Maesawa C, Tamura G and Satodate R (1995) Frequent microsatellite alterations on chromosome 3p in esophageal squamous cell carcinoma. *Cancer Res* **55**: 891–894

Ogasawara S, Tamura G, Maesawa C, Suzuki Y, Ishida K, Satoh N, Vesugi N, Saito K and Satodate R (1996) Common deleted region on the long arm of chromosome 5 in esophageal carcinoma. *Gastroenterology* **110**: 52–57

Ohta M, Inoue H, Cotticelli MG, Kastury G, Baffa R, Palazzo J, Siprashvili Z, Mori M, McCue P, Druck T, Croce CM and Huebner K (1996) The FHIT gene, spanning the chromosome 3p14.2 fragile site and rural carcinoma-associated t (3; 8) breakpoint, is abnormal in digestive tract cancers. *Cell* **84**: 587–597

Porschen R, Bevers G, Remy U, Schauseil S and Borchard F (1993) Influence of preoperative radiotherapy on DNA ploidy in squamous cell carcinomas of the oesophagus. *Gut* **34**: 1086–1090

Powell SM, Papadopoulos N, Kinzler KW, Smolinski KN and Meltzer SJ (1994) APC gene mutations on the mutation cluster region are rare in oesophageal carcinoma. *Gastroenterology* **107**: 1759–1763

Reid BJ, Haggitt RC, Rubin CE, Roth G, Surawicz CM, Van Belle G, Levin K, Weinstein WM, Antonioli DA, Goldman H, McDonald W and Owen D (1988) Observer variation in the diagnosis of dysplasia in Barrett's esophagus. *Hum Pathol* **19**: 166–178

Schnell T, Sontag SJ, Chejfec G, Kurucar C, O'Connell S, Levine J, Karp K, Adelman S and Reid L (1996) High-grade dysplasia is not an indication for surgery in patients with Barrett's esophagus. *Gastroenterology* **110**: A590

Shibagaki I, Shimada Y, Wagata T, Ikenaga M, Imamura M and Ishizaki K (1994) Allelotype analysis of esophageal squamous cell carcinoma. *Cancer Res* **54**: 2996–3000

Shimizu T and Sekiya T (1996) Loss of heterozygosity at 9p21 loci and mutations of the MTS1/p16 and MTS2 genes in human lung cancers. *Int J Cancer* **63**: 515–520

Sozzi G, Veronese ML, Negrini M, Baffa R, Cotticelli MG, Inoue H, Tornielli S, Pilotti S, De-Gregorio L, Pastorino I, Pierotti MA, Ohta M, Huebner K and Croce M (1996) The FHIT gene is abnormal in lung cancer. *Cell* **85**: 17–26

Spechler SJ (1987) Endoscopic surveillance for patients with Barrett's esophagus: does cancer risk justify the practice? *Ann Int Med* **106**: 902–904

Spechler SJ, Robbins AH, Rubins RB, Vincent ME, Hereen T, Doos WG, Colton T and Schimmel EM (1984) Adenocarcinoma and Barrett's oesophagus: an overrated risk? *Gastroenterology* **87**: 927–933

Spechler SJ, Zeroogian JM, Antonioli DA, Wang HH and Goyal RK (1994) Prevalence of metaplasia at the gastro-oesophageal junction. *Lancet* **344**: 1533–1536

Swift A, Risk JM, Kingsnorth AN, Wright TA, Myskow M and Field JK (1995) Frequent loss of heterozygosity on chromosome 17 at 17q11.2–q12 in Barrett's adenocarcinoma. *Br J Cancer* **71**: 995–998

Van den Berg A, Hulsbeek MF, de Jong D, Kok K, Veldhuis PM, Roche J and Buys CH (1996) Frequent LOH on chromosome 9 in adenocarcinoma and squamous cell carcinoma of the oesophagus. *Genes Chromosom Cancer* **15**: 64–72

Vogelstein B, Fearon ER, Kern SE, Hamilton SR, Preisinger AC, Nakamura Y and White R (1989) Allelotype of colorectal carcinomas. *Science* **244**: 207–211

Waber PG, Lee NK and Nisen PD (1996) Frequent allele loss at chromosome arm 3p is distinct from genetic alteration in the von Hippel Lindau tumour suppressor gene in head and neck cancer. *Oncogene* **12**: 365–369

Wagata T, Ishizai K, Imamura M, Shimada Y, Ikenaga M and Tobe T (1991) Deletion of 17p and amplification of the int-2 gene in esophageal carcinomas. *Cancer Res* **51**: 2113–2117

Walsh TN, Dorman AM, Droogan O, Curran B, Hourihane D, Leader M and Hennessy TB (1992) DNA ploidy in squamous oesophageal carcinoma. *Surg Oncol* **1**: 37–42

Williamson WA, Ellis FH, Gibb P, Shahian DM, Aretz HT, Heatley GJ and Watkins E (1991) Barrett's esophagus: prevalence and incidence of adenocarcinoma. *Arch Int Med* **151**: 2212–2216

Yamaguchi T, Toguchida J, Yamamuro T, Kotoura Y, Takada N, Kawaguchi N, Kaneko Y, Nakamura Y, Sasaki S and Ishizaki K (1992) Allelotype analysis in osteosarcomas: frequent allele loss on 3q, 13q, 17p and 18q. *Cancer Res* **52**: 2419–2423

Zhou X, Tarmin L, Yin J, Jiang HY, Suzuki H, Rhyu MG, Abraham JM and Meltzer SJ (1994) The MTS1 gene is frequently mutated in primary human esophageal tumours. *Oncogene* **9**: 3737–3741

Zhuang Z, Emmert-Buck MR, Roth MJ, Gnarra J, Linehan WM, Liotta LA and Lubensky IA (1996) Von Hippel Lindau disease gene deletion detected in microdissected sporadic human colon carcinoma specimens. *Hum Pathol* **27**: 152–156

© Cancer Research Campaign 1998